# Occurrence of *Vibrio* and *Salmonella* species in mussels (*Mytilus galloprovincialis*) collected along the Moroccan Atlantic coast

**DOI:** 10.1186/2193-1801-3-265

**Published:** 2014-05-24

**Authors:** Hasna Mannas, Rachida Mimouni, Noureddine Chaouqy, Fatima Hamadi, Jaime Martinez-Urtaza

**Affiliations:** Faculty of Sciences, Laboratory of Biotechnology & Valorisation of Natural Resources, University Ibn Zohr, Agadir, Morocco; Laboratory of Microbiology, National Health Security Food Office (ONSSA), Agadir, Morocco; Reader in Infection and Immunology Department of Biology and Biochemistry, University of Bath, BA2 7AY Bath, United Kingdom

**Keywords:** *Salmonella*, *Vibrio*, *Escherichia coli*, *Mytilus galloprovincialis*, Agadir, Essaouirra, Morocco, Mussels

## Abstract

This study reports the occurrence of different *Vibrio* and *Salmonella* species in 52 samples of *Mytilus galloprovincialis* collected from four sites along the Atlantic coast between Agadir and Essaouira (Anza, Cap Ghir, Imssouane and Essaouira). The level of *Escherichia coli (E. coli)* was also determined to evaluate the degree of microbial pollution in the investigated areas. In this study three methods were used : AFNOR NF EN ISO 6579 V08-013 for *Salmonella* spp., the provisional method routinely used by several laboratories (Institut Pasteur, Paris,…) for *Vibrio cholerae* and *Vibrio parahaemolyticus* in the seafood, and the most probable number method (MPN) using Norm ISO/TS 16649–3 (2005) for *E. coli*. The most frequently isolated *Vibrios* were *Vibrio alginolyticus* (90.4% of samples), followed by *V. cholerae* non O1 non O139 (15.4%) and *V. parahaemolyticus* (7.7%). *Salmonella* spp. was found in 15% of the samples. The number of *E. coli* ranged between 0.2/100 g and 1.8 10^3^ /100 g of mussel soft tissues. This study indicates the potential sanitary risk associated with the presence of pathogenic bacteria in cultivated mussels in the two populous regions of southern Morocco, where shellfish production and maritime tourism are important to the local economy.

## Introduction

Bivalve shellfish are marine invertebrates known to be reliable indicators of pollution in marine environment (Goldberg et al. 1978; Cossa 1989; Ramade 1992). In fact, they present characteristics of bioindicator organisms because they have the capacity to accumulate microorganisms from surrounding waters because of their filter-feeding strategy (Desenclos et al. 1996). Shellfish especially mussels, may be a vehicle for most known pathogen bacteria (Huss 1997). Non-indigenous pathogen bacteria (e.g. *Salmonella* and *Shigella*) are introduced into seawater by infected animals and humans, while indigenous bacteria are natural occurring organisms in the marine environment, mainly belonging to the family *Vibrionaceae* (Potasman et al. 2002). Therefore, these bacteria constitute a potential risk for consumers. Infections were due, either to the consumption of raw or inadequately cooked bivalves, or improperly processed shellfish (FDA 2004). The genus *Vibrio* is endemic in marine and estuarine ecosystems, and regroups more than 63 species. Since many of them were pathogenic for humans, they had been associated with frequent food-borne infections (Chakraborty et al. 1997). Among these species, 12 might cause gastrointestinal diseases or, in some cases, septicemia. Most of them were caused by *Vibrio parahaemolyticus* and *V. vulnificus* (Oliver and Kaper 1997).

Several cases of gastroenteritis, due to the consumption of sea products, were listed each year in Morocco (Cohen and Karib 2007) and their etiology was often not known. They might be caused by these *Vibrios* or any other enteric pathogen or toxin (Cohen and Karib 2007). On the other hand, it had been reported that all the *Vibrio* collective food poisoning infection in Morocco were due to the consumption of bivalves clandestinely collected (Bouchriti et al. 2001). The prevalence of these *Vibrios* in Moroccan seafood was already reported by Bouchriti et al. (2001).

In Morocco, few studies (Bouchriti et al. 2001; Cohen and Karib 2007; Setti et al. 2009) have been done on the occurrence of the pathogenic bacteria in the seafood in order to better estimate the prevalence of these pathogens in the marine environment. Such studies are necessary to carry out the analysis of risk associated with these pathogens to reduce the manifestation of collective food poisoning infection. The first aim of this study was to investigate the occurrence of *Vibrio* and *Salmonella* in *Mytilus galloprovincialis* along the Moroccan Atlantic coast between Agadir and Essaouirra. The second aim was to determine the existence of any correlation between the occurrence of *Vibrio* spp. and *Escherichia coli* (indicator bacteria).

## Materials and methods

### Study area

**Figure 1:**
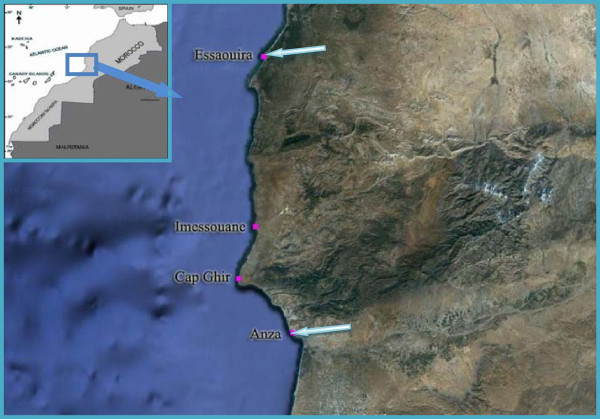
**The four mussel beds investigated along the Atlantic coast from April 2010 until April 2011 (Arrows indicate the location of wastewater discharges).**

This area is located along the West Atlantic coast of Morocco which extends from Anza to Essaouirra and has a length of approximately 150 km (Figure 1). This coast is characterized by many natural beds of mussels (*M. galloprovincialis*). This work was realized in two bays (Agadir and Essaouirra). Agadir is located in West Central Morocco between approximately 9° 13′10 “and 9° 53′30” degrees West longitude and 30° 56′00 “and 30° 20′50” north latitude.

Agadir is characterized by a semiarid climate with a cool and a warm season. The average temperature between 2000 and 2012 is 26°C (21°C - 31°C). The temperature can reach over 40°C under the influence of high saharien winds. The average of rainfall between 2000 and 2012 is 243 mm/year. The rainy season mainly occurs in the fall and in the first weeks of winter according to data obtained from the Regional Office of the Development of Agricultural Souss Massa. Coastal waters of Agadir bay are influenced by two types of currents: the Canary current, moving to the southwest at a speed of 0.5 knots (0.5 nautical mile/h). The second is the Upwelling characterized by deep water rising to the website surface resulting in high concentrations of nutrients especially in summer (Mimouni et al. 2003; Mimouni, 2004; Orbi, 1991).

Essaouirra is a city in the western Moroccan. The climate of Essaouirra is arid with a dry period of six to seven months. The average temperature of Essaouirra is 16.7°C, with a relatively small difference between the average temperatures of the warmest and the coolest month. Essaouirra is characterized by a rainy season from October to May and an average dry season (June-September). The average rainfall is 280 mm/year. It’s also characterized by the influence of the Upwelling of cold water along the coast by the predominance of maritime origin strong winds throughout the year. Strong winds are constant at Essaouira.

Four sites were selected for this study: i) Anza (30° 26′ N, 9° 38′ W) which receives discharges of domestic sewage and industrial untreated waters; ii) Cap Ghir (30° 0′ N, 9° 53′ W) characterized by the presence of natural deposit and a major tourism activity. We considered this site as site reference; iii) the Imsouane bay (30° 50′.46″ N, 9° 46′.44″ W) is located at 100 km from Agadir; this site corresponding to the mouth of the Tablast river, is known to be a center for speedboaters, canoeists and fishing; iv) Essaouirra (31° 54′ N, 9° 76′ W) subjected to a great environmental pollution (discharge of sewages and garbage).

### Seawater physicochemical parameters

Temperature, salinity and pH were measured along the sampling period (April 2010-April 2011). The temperature of seawater was measured using a laboratory thermometer. The pH was measured by a pH-meter (WTW pH 522), and values of salinity were detected using a salinometer (W.T.W.LF18, Measuring Cells Tetracon 325).

### Sample collection and preparation

Every month, 52 mussel samples were collected from the four sampling sites between April 2010 and April 2011 to detect the presence of *Vibrios* and *Salmonellae*. Each sample consisted of 30 specimens of *M. galloprovincialis*. These bivalves were placed into sterile bags and transported to the laboratory under a temperature of 4°C. All samples were examined within two hours following their collection. At their arrival in the laboratory, the mussels were scrubbed under running tap water to remove shell debris and attached algae. They were then dried and aseptically opened using a sterilized scalpel.

### Isolation and biochemical identification of Vibrios

*Vibrio* isolation was performed with a provisional method routinely used by several laboratories: National Reference Center for Vibrios and Cholera, Institut Pasteur, Paris, France; Laboratory of Studies and Research in Environment and Health, National School of Public Health, Rennes, France; Laboratory for Studies and Research on Fishery Products, AFSSA-Site of Boulogne, Boulogne-sur-Mer, France.

A 1:9 dilution was first prepared with 25 g of soft tissue and intervalvular liquid in 225 mL of Alkaline peptone water (APW) (Yeast extract 3 g, peptone 10 g, sodium chloride 20 g/L and distilled water 1 L at pH 8.6). The mix was homogenized and incubated at 42 °C for 18 h for its enrichment and cell growth. An aliquot was innocullated in Thiosulfate citrate bile sacharose agar (TCBS) (Scharlau Chimie, Spain) and CHROMagar *Vibrio* chromogenic medium (Oxoid, Wesel, Germany). These media were then incubated at 37°C for 18–24 h. A minimum of five typical colonies were picked and cultured on Marine agar to determine their purity (ZoBell 1941). The pure cultures of isolated colonies were subjected to a battery of tests for morphological and biochemical identification. Strains were identified by Gram staining and cytochrome oxidase activity. Only Gram-negative, oxidase-positive colonies were selected for biochemical tests, following classical procedures. The API 20E system, and culture in 0%, 3%, 6%, 8% and 10% NaCl peptone water were used to identify the *Vibrio* species (Hara-Kudo et al. 2001). Serotyping strains of *V. cholerae* consisted of a slide agglutination test with a 24 hour culture on Marine Agar (MA), a test in physiological saline was performed to verify that the strains were not autoagglutinables. The Suspected colonies resembling *V. cholerae* were tested by slide agglutination with polyvalent anti-O1 and anti-O139 sera.

### Isolation and biochemical identification of Salmonella

These bacteria were isolated according to the norm ISO 6579 (2002). Briefly, 225 mL of Buffered peptone water was added to 25 g of homogenized mussel soft tissues and incubated at 37°C for 20 h. After this pre-enrichment step, 0.1 mL of the solution was transferred in 10 mL of the Rappaport-vassiliadis Broth (Scharlau Chemie, Spain) and the mix was incubated at 41,5°C for 24 h. Other 1 mL were added to 10 mL of the Muller-Kauffman au Tetrathionate-Novobiocine (MKTTn) (Scharlau Chemie, Spain) and were incubated at 37°C for 24 h. The enriched suspensions were then plated on Rambach Agar (Merck) and on Xylose Lysin Desoxycholate Agar (Scharlau Chemie, Spain), and were incubated at 37°C for 24 h. Suspect colonies were purified with Tryptone Sulfite Neomycin agar (TSN) (Scharlau Chemie, Spain), and confirmed biochemically with the API 20 E system (bioMérieux, Marcy-l’Étoile, France).

### Enumeration of Escherichia coli

The most probable number (MPN) of *E. coli* in samples was determined using the multiple tube method with the 5-tubes-3-dilutions test according to the norm ISO/TS 16649–3 (2005). Seventy-five grams of flesh and intervalvar liquid (FIL) were diluted 1:3 with the Tryptone salt water (biokar Diagnostics) and the mix was homogenized using a food homogenizer (Stomacher) for 2 min to obtain a stock suspension. Thirty mL of this mix were added to 70 mL of Tryptone salt water (biokar Diagnostics) and were homogenized to reach a 1:10 dilution. Ten mL of this homogenate were inoculated into five tubes of double concentrate of Minerals Modified Glutamate Broth (MMGB). Five tubes of normal concentration MMGB were inoculated with 1 mL of the 1:10 homogenate, while the other five tubes were inoculated with 1 mL of a 1:100 dilution per tube. After homogenizing, all the tubes were incubated at 37°C for 24 h. *Escherichia coli* confirmation was performed by culturing 1uL of positive tubes (tubes showing acid production) on the Tryptone Bile X-Glucuronide agar (TBX) (Oxoid, Wesel, Germany), and by incubating them at 44°C for 24 h. The growth of blue colonies indicated the presence of *E. coli.* The number of *E. coli* per 100 g was determined using MPN tables (ISO/TS 16649–3 (2005)) according to the number of positive results in the three dilutions.

### Statistical analysis

After all parameters were considered; to evaluate if there are any correlation between these parameters, Principle Components Analysis was conducted by Statistica software V6.

## Results

### Isolation and biochemical identification of Vibrios

Among the 52 samples examined, *Vibrio* spp*.* was found in the four sites, with percentages ranging from 7.7% to 100% (Table 1). The use of two agar media (TCBS and the CHROMagar *Vibrio* chromogenic medium) in the present study to isolate the *Vibrio* spp. influence the detection of higher number of positives samples. Our findings suggest that the CHROMagar *Vibrio* medium was more efficient and accurate for identifying *Vibrio* spp. in mussels than the conventional TCBS agar. Otherwise serological analysis strains identify *V. cholerae* shows no agglutination with the serum anti-O1 and anti-O139 serum.

**Table 1:** **Prevalence of positive samples of mussels for** ***Vibrio*** **spp. and** ***Salmonella*** **spp. for each sites studied between April 2011 and April 2012**

Sites	***V. alginolyticus***	***V. cholerae***	***V. parahaemolyticus***	***Salmonella***
Anza	76.9 (10/13)*	23.1 (3/13)	15.4 (2/13)	38.5 (5/1 3)
Cap Ghir	92.3 (12/13)	7.7 (1/13)	0 (0/13)	0 (0/13)
Imssouane	92.3 (12/13)	7.7 (1/13)	7.7 (1/13)	7.7 (1/13)
Essaouira	100 (13/13)	23.1 (3/13)	7.7 (1/13)	15.4 (2/13)
All sites	90.4 (47/52)	15.4 (8/52)	7.7 (4/52)	15.4 (8/52)

*(a/b): (a = Number of positive samples / b = Total number of samples analyzed for each site).

**Figure 2:**
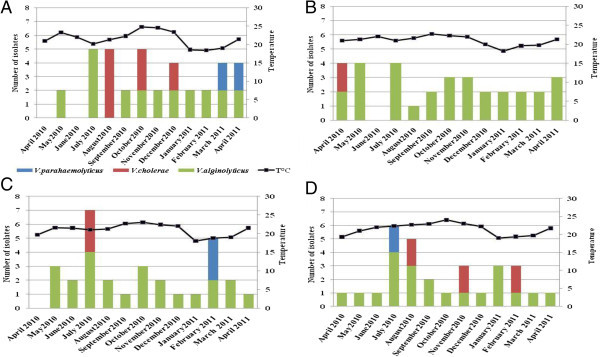
**Monthly variations of seawater temperature and number of** ***Vibrio*** **isolates containing in mussels in the four sites: Anza (A), Cap Ghir (B), Imssouane (C) and Essaouira (D).**

*V. alginolyticus* was detected in 90.4% of the samples collected at the four sites during all the period of our study (Table 1). *V. cholera* non O1 non O139 was detected in 15.4% of the samples (Table 1). This bacterium is very abundant in August, November and February, in the Essaouirra site (Figure 2D). Also in Anza site this pathogen germ was isolated between August and December (Figure 2A). The prevalence of this bacterium was lower, it was found only in April and July in Cap Ghir (Figure 2B) and Imssouane respectively (Figure 2C).

For *V. parahaemolyticus* 15.4% and 7.7% of samples were contaminated in Anza and Essaouirra respectively (Table 1). Generally, it was found in July in the Essaouirra site (Figure 2D) and February in Imssouane site (Figure 2C). For the other sites, this pathogen germ was almost absent in Cap Ghir (Figure 2B), but at Anza site this bacteria was observed at March and April (Figure 2A).

**Figure 3:**
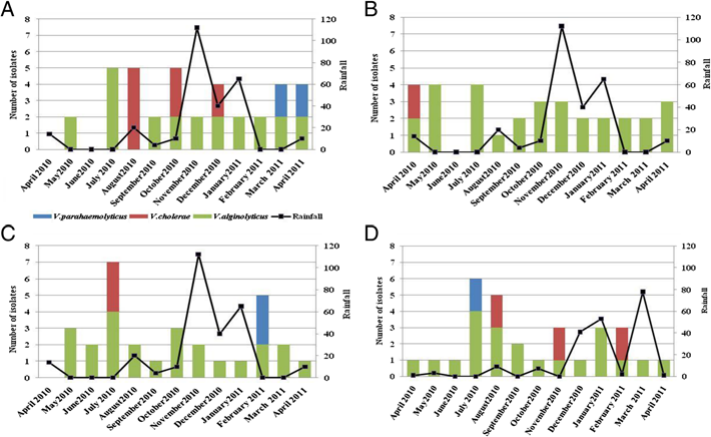
**Relationship between number of** ***Vibrio*** **isolates and rainfall in the sites studied Anza (A), Cap Ghir (B), Imssouane (C) and Essaouira (D).**

*V. alginolyticus*, *V. parahaemolyticus* and *V. cholerae* non O1 non O139 represented the found species in the four sites studied. *V. alginolyticus* was the most abundant species recovered from all the samples. The variation of number of *Vibrio* isolate with rainfall was presented in Figure 3. We observed that the number of *V. cholerae* non O1 non O139 and *V. parahaemolyticus* isolate decreased with rainfall in all sites and particularly in Anza (Figure 3A) and Essaouira (Figure 3D).

### Occurrence of Salmonella

**Figure 4:**
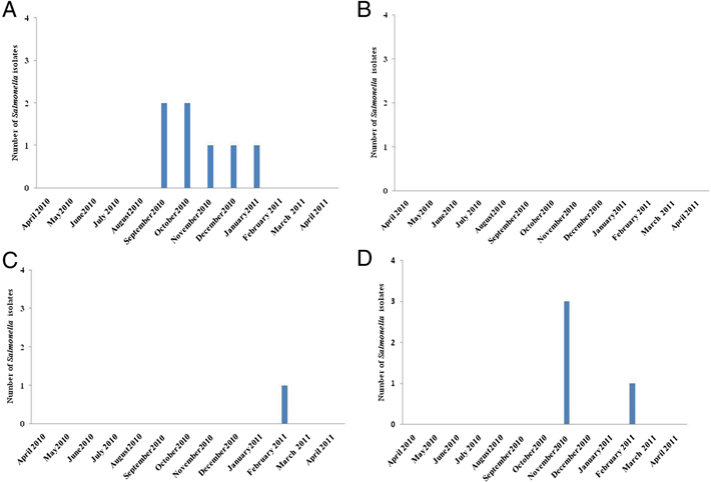
**Number of** ***Salmonella*** **spp. isolates containing in mussels in the four sites: Anza (A), Cap Ghir (B), Imssouane (C) and Essaouira (D).**

**Figure 5:**
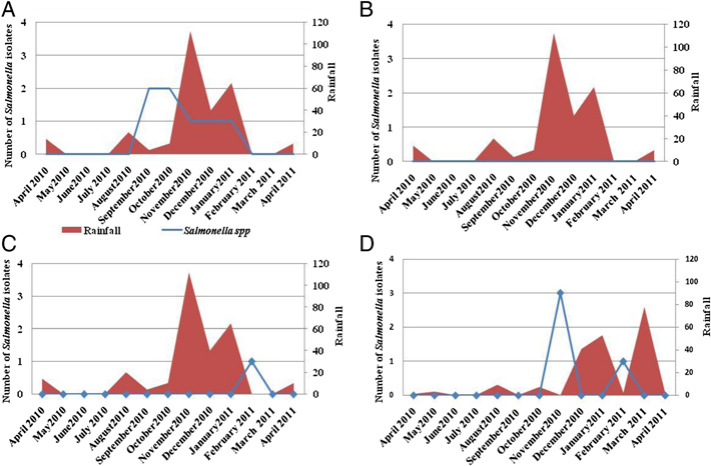
**Relationship between number of** ***Salmonella*** **isolates and rainfall in the sites studied (Anza: A, Cap Ghir: B, Imssouane: C and Essaouira: D).**

In the present study, *Salmonella* spp. was recovered from 15.4% of mussel samples (Table 1). The eight positive samples were collected from three sites (Anza, Imssouane and Essaouira) (Figure 4). The highest prevalence of *Salmonella* was noted in the samples collected from Anza (38.5%) (Table 1). *Salmonella* was predominantly detected during the rainy and cool season between September and January (Figure 4A). In Essaouira site *Salmonella* was found in 15.4% of samples (Table 1), this bacterium is abundant in November and February (Figure 4D). The lower prevalence of *Salmonella* was registered in Imssouane site where only one positive sample was detected in February (Figure 4C). The relation between the number of *Salmonella* spp and rainfall was presented in Figure 5. No relation was observed between the presence of *Salmonella* spp. and rainy periods.

### *Escherichia coli* enumeration

**Figure 6:**
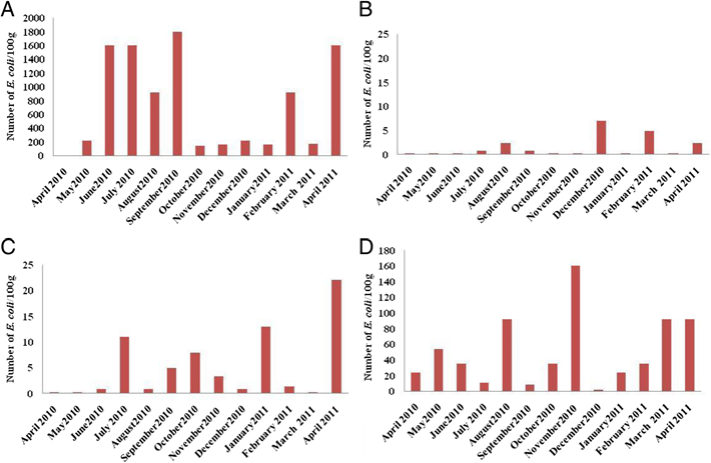
**Number of** ***E. coli*** **/100 g containing in mussels in the sites studies: Anza (A), Cap Ghir (B), Imssouane (C) and Essaouira (D).**

The number of *E coli* detected in Anza ranged from 1.4 10^2^/100 g to 1.8 10^3^/100 g (Figure 6A). The highest evels of *E. coli* were detected during the dry season. In Essaouirra, the lowest (2.2/100 g) and highest (1.6 10^2^/100 g) *E. coli* counts were found in December and November respectively (Figure 6D). The maximum level of *E. coli* was observed in December when rainfall was present few days before sampling at Cap Ghir (Figure 6B) whereas it was obtained in April at Imssouane site (Figure 6C).

**Figure 7:**
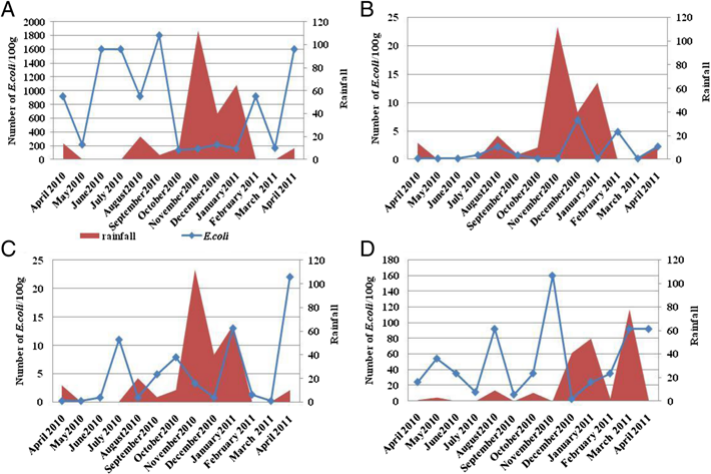
**Relationship between number of** ***E. coli*** **isolates and rainfall in the sites studied: Anza (A), Cap Ghir (B), Imssouane (C) and Essaouira (D).**

Statistical analysis was performed by the principal component analysis (PCA) which shows that there is no correlation between the presence of pathogenic *Vibrios* and *Salmonella* spp. in mussels and the presence of the *E. coli* contamination. The results of *E. coli* concentration variation with rainfall are illustrated in Figure 7. These results show that the concentration of *E. coli* was mostly limited to the rainy period in all sites.

### Principal component analysis

**Figure 8:**
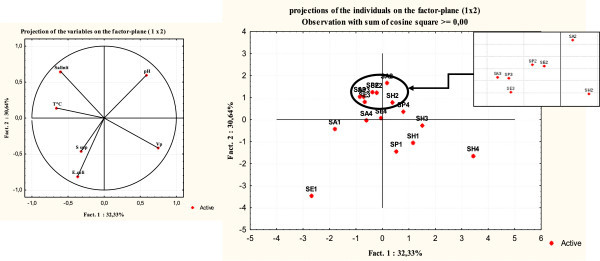
**Principal Component Analysis concerning the parameters physicochemical and bacteriological at the four sites studied. Spp**: S*almonella* spp; **Vp**: *Vibrio parahaemolyticus*; **T**°: Temperature; ***E*** . ***coli***: *Escherichia coli*; **SP1**: Anza spring; **SE1**: Anza summer; **SA1**: Anza autumn; **SH1**: Anza winter; **SP2**: Cap Ghir spring; **SE2**: Cap Ghir summer; **SA2**: : Cap Ghir autumn; **SH2**: Cap Ghir winter ; **SP3**: Imssouane spring; **SE3**: Imssouane summer; **SA3**: Imssouane autumn; **SH3**: Imssouane winter; **SP4**: Essaouira spring; **SE4** : Essaouira summer; **SA4**: Essaouira autumn.

The principal component analysis (PCA) was performed on the physicochemical of seawater (Table 2) and bacteriological variable of mussels collected in the four sites (Table 3). The projection of the variables on the two axes of the PCA indicates that the two axes of the PCA represented 62.97% of the total inertia. Circle correlation of variables showed that *V. parahaemolyticus* and pH are positively correlated with respect to the axis 1. By one against the temperature correlated negatively with respect to the axis 1. Thus, the variables *E. coli* and *Salmonella* spp are negatively correlated with axis 2 against salinity correlates positively with axis 2. In addition, the two variables salinity and *V. parahaemolyticus* systematically oppose against axis 2. The number of isolates of *V. parahaemolyticus* is very important in the Anza site and has low salinity values in reports to site Cap Ghir (Figure 8A). The two axes of the PCA factorial account 62.97% of the total inertia (Figure 8B). Therefore two different groups are distinguished. A first group gathering sites 2 and 3 corresponding to the Cap Ghir and Imssouane, the second gathering sites 1 and 4 corresponding to Anza and Essaouirra sites. The comparison of the two PCA (Circle correlation of variables and factorial PCA) shows that the first group has a strong correlation with salinity. This indicates salinity is very important in the waters of Cap Ghir and Imssouane sites. The second group is divided into three subgroups. A first subgroup represented by the site of Essaouira and the Anza site with three seasons. This subgroup is strongly correlated with *Salmonella* spp., it shows that the mussels taken from these two sites present a high concentration of these bacteria. The second subgroup is represented solely by the Anza site summer season and strictly correlates with *E. coli.* Mussels collected on this site have high rates of *E. coli* during the summer. The third group presents Essaouirra during the winter season it is strongly correlated with *V. parahaemolyticus* indicates that this organism is very important in mussels collected during this period.

**Table 2:** **Correlation matrix obtained from the principal component analysis of physicochemical and bacteriological parameters of mussels collected from the study sites**

Parameter	T°C	pH	Salinity	***V. p.***	***S***. spp.	***E. coli***
T°C	1	−0.062478	0.320826	0.255041	0.363347	0.036097
pH	−0.062478	1	−0.099246	0.181816	−0.154248	−0.710888
Salinity	0.320826	−0.099246	1	−0.508414	−0.300831	−0.196118
*V. p*.	0.255041	0.181816	−0.508414	1	0.258185	0.054976
*S.* spp.	0.363347	−0.154248	−0.300831	0.258185	1	0.233055
*E. coli*	0.036097	−0.710888	−0.196118	0.054976	0.233055	1

V. p.: Vibrio paraheamolyticus, S. spp.: Salmonella spp., E. coli: Escherichia coli, T°: Temperature.

**Table 3:** **Annual averages of physicochemical parameters**

Sites	Temperature* (Min-Max)	pH* (Min-Max)	Salinity* (Min-Max)
Anza	21.6	7.5	35.2
	18.6-24,8	6.7-8	31.6-36.1
Cap Ghir	21.0	7.8	36.4
	18.2-22.7	7.6-8.1	35.9-37.1
Imssouane	21.0	7.6	36.4
	18-23	7.4-7.9	37.2-34.3
Essaouirra	21.5	7.7	34.8
	19-24	7.3-7.9	30-36

*The value is the average of thirteen values (April 2010-April 2011).

The examination of correlation coefficients between the variables (Table 3) shows that the pH has a significant and negative correlation with *E. coli* (r = −0.71).

## Discussion

The major objective of this study was to determine the prevalence of potentially pathogenic *Vibrio* spp., *Salmonella* spp*.* and *E. coli* in mussel (*M. galloprovincialis*), collected from Agadir and Essaouira coastal areas that vary in water temperature and salinity. *Vibrio* spp. was found in all the sites, with percentages ranging from 7.7% to 100%.

Compared to the other reports which investigated the mussels (*M. edulis* and *M. galloprovincialis*), the percentage of *Vibrio*-positive samples in the present study was higher than the occurrence found in previous studies carried out in Germany (Wadden Sea: 74.4%), Ionian (Mar Piccolo of Taranto Sea: 60%) and Italy (Adriatic Sea: 48.4%) (Lhafi and Kuhne 2007; Cavallo and Stabili 2002; Ripabelli et al. 1999). The difference obtained between our results and these reports may be associated with the existence of different climate conditions in the different areas. Additionally, the use of two agar media (TCBS and the CHROMagar *Vibrio* chromogenic medium) in the present study to isolate the *Vibrio* spp., may also influence the detection of higher number of positives samples with respect to previous studies (e.g., Lhafi and Kuhne (2007) and Ripabelli et al. (1999)) which used TCBS medium only. CHROMagar *Vibrio* medium contains substrates for beta-galactosidase. It was specifically developed to differentiate *V. parahaemolyticus* from *V. vulnificus*, *V. cholerae* non O1 non O139 and other Vibrios directly at the isolation step on colony color by using a chromogenic substrate (instead of sugar fermentation in traditional growth media such as TCBS). On the CHROMagar *Vibrio* medium, *V. parahaemolyticus* colonies were purple. Those of *V. vulnificus* and *V. cholerae* appeared blue, while *V. alginolyticus* colonies were colorless. Our findings suggest that the CHROMagar *Vibrio* medium was more efficient and accurate for identifying *Vibrio* spp. in mussels than the conventional TCBS agar. This result agreed with the reports of Di Pinto et al. (2011), Croci et al. (2007) and Blanco-Abad et al. (2009).

*V. alginolyticus, V. parahaemolyticus* and *V. cholera* non O1 non O139 represented the found species in the four sites studied. *V. alginolyticus* was the most abundant species recovered from all the samples. In fact, seawater is the normal habitat for *V. alginolyticus* and was isolated from seawater and seafood in many parts of the world. The study of Matté et al. (1994) reported that the *V. alginolyticus* was the most common *Vibrio* species found in the *Perna perna* from the Atlantic coastal of Brazil, furthermore Ripabelli et al. (1999) found similar results for the *M. galloprovincialis* harvested from Adriatic sea in Italy. These results were in agreement with the reports by Cavallo et al. in Mar Piccolo of Taranto (Ionian Sea) (2002), Hervio-Heath et al. (2002) in coastal areas of France, and Covazzi Harriague et al. (2008) in northern Adriatic Sea (Italy) who studied the *M. galloprovincialis*, mussels (*M. edulis and M. galloprovincialis*), and sedimentary crustaceans (copepods and anphipods) respectively.

The number of isolates of *Vibrio* especially *V. cholerae* non O1 non O139 and *V. parahaemolyticus* in the study site particularly in Anza and Essaouira decreased with rainfall. The result of this study is in contrast to the results of Yamazaki and Esiobu (2012) who found a correlation between the rainfall dates and the incidence of pathogenic *Vibrio* species.

Except Cap Ghir site, *Salmonella* spp. was found in all sites. The highest prevalence (38.5%) of *Salmonella* was noted in the samples collected from Anza. Our results are similar to several reports (Brands et al. 2005; Setti et al. 2009; Bakr et al. 2011). Brands et al. (2005) detected the presence of these bacteria in oysters (7.4%) from different coasts of the United States. Setti et al. (2009) reported the prevalence of *Salmonella* in 10% of 801 samples of mussels collected from Agadir coast. Bakr et al. (2011) found this bacterium in 8.0% of their samples (Alexandria, Egypt). In a recent study by Boutaib et al. (2011) carried out in Morocco (Rabat) on faecal contamination using 104 samples of Callista chione and Acanthocardia tuberculatum, indicated that the presence of *Salmonella* was detected in 7 samples of A. tuberculatum and two of C. chione.

Our results show that the presence of *salmonella* was reduced in rainy periods. These findings were in agreement with Simental and Martinez-Urtaza (2008) who found that the presence of *Salmonella* spp. in coastal areas was mostly confined to rainy periods. On the other hand our results were in contrast with reports of Martinez-Urtaza et al. 2008 and Setti et al. 2009, who reported that the incidence of *Salmonella* spp. was associated with high rainfall.

*E. coli* was detected in Anza with values ranging from 1.4 10^2^/100 g and 1.8 10^3^/100 g. The highest levels of *E. coli* were detected during the dry season. In Essaouirra, the lowest and highest *E. coli* counts were noted in December and November respectively. The maximum load of *E. coli* was observed in December when rainfall was present few days before sampling. Higher levels of *E. coli* were detected throughout almost all the months of the year at Anza and Essaouirra. Indeed, these sites received continuous discharges coming from domestic and industrial wastewater without any prior treatment (Mimouni 2004, 2002). The variation of the concentration of *E. coli* with rainfall shows that the number of *E. coli* was mostly limited to the rainy period in all sites. These findings were in accordance with the work of Kleinheinz et al. (2009). In this work the authors found that the concentration of *E. coli* decreases with rainfall.

Statistical analysis (PCA) shows that there is no correlation between the presence of pathogenic Vibrios and *Salmonella* spp. in mussels and the presence of the *E. coli* contamination. This is consistent with the findings of other authors (Matté et al. 1994; Normanno et al. 2006). In the study of Boutaib et al. 2011, the faecal contamination (counts of *E. coli*) was high through the year and above of the threshold 230 *E. coli/*100 g in both bivalves harvested from Fnideq location (Morocco).

he principal components analysis (PCA) was performed on the physicochemical of seawater and bacteriological variable of mussels collected in all the sites investigated. The result indicates that *V. parahaemolyticus* and pH are positively correlated with respect to the axis 1. *E. coli* and *Salmonella* spp. are negatively correlated with axis 2 against salinity correlates positively with axis 2. In addition, the two variables salinity and *V. parahaemolyticus* systematically oppose against axis 2. The PCA analysis distinguishes two different groups. A first group gathering sites 2 and 3 corresponding to the Cap Ghir and Imssouane, the second gathering sites 1 and 4 corresponding to Anza and Essaouirra sites. The statistical analysis revealed that there was a negative correlation between the presence of *V. parahaemolyticus* and salinity. In Alabama Gulf of Mexico, Johnson et al. (2010) reported that there was no relation between salinity and *Vibrio* densities. In contrast, in Mississippi, a significant positive relationship with salinity was identified (Johnson et al. 2010). This is due to the fact that the salinity is not the same at both sites. Other studies have also identified the importance of salinity to Vibrios densities (Motes et al. 1998; Lipp et al. 2001; Martinez-Urtaza et al. 2008). The salinity ranges in these studies varied, but all were narrower.

In conclusion, the results noted in the present study showed the *V. alginolyticus* was the predominant *Vibrio* spp. in the four sites of sampling; additionally, it was also denoted that faecal contamination was high in Anza and Essaouira with a significant prevalence of *Salmonella* in both sites. Fecal contamination may have been introduced into the coastal by wastewaters and rainfall. Finally, no relationship was established between the presence of *Vibrio* spp., *Salmonella* spp. and fecal pollution, which represents additional evidence indicating that the presence of *Vibrio* in the area is a natural event.
